# Tongue coating microbiome as a potential biomarker for gastritis including precancerous cascade

**DOI:** 10.1007/s13238-018-0596-6

**Published:** 2018-11-26

**Authors:** Jiaxing Cui, Hongfei Cui, Mingran Yang, Shiyu Du, Junfeng Li, Yingxue Li, Liyang Liu, Xuegong Zhang, Shao Li

**Affiliations:** 1 MOE Key Laboratory of Bioinformatics and TCM-X center/Bioinformatics Division, BNRist/Department of Automation, Tsinghua University, Beijing, 100084 China; 2 China-Japan Friendship Hospital, Beijing, 100029 China; 3 Institute for Artificial Intelligence and Department of Computer Science and Technology, Tsinghua University, Beijing, 100084 China; 4 School of Life Sciences and Center for Synthetic and Systems Biology, Tsinghua University, Beijing, 100084 China

**Keywords:** gastritis, tongue coating, metagenomics, *Campylobacter concisus*, non-invasive biomarker

## Abstract

The development of gastritis is associated with an increased risk of gastric cancer. Current invasive gastritis diagnostic methods are not suitable for monitoring progress. In this work based on 78 gastritis patients and 50 healthy individuals, we observed that the variation of tongue-coating microbiota was associated with the occurrence and development of gastritis. Twenty-one microbial species were identified for differentiating tongue-coating microbiomes of gastritis and healthy individuals. Pathways such as microbial metabolism in diverse environments, biosynthesis of antibiotics and bacterial chemotaxis were up-regulated in gastritis patients. The abundance of *Campylobacter concisus* was found associated with the gastric precancerous cascade. Furthermore, *Campylobacter concisus* could be detected in tongue coating and gastric fluid in a validation cohort containing 38 gastritis patients. These observations provided biological evidence of tongue diagnosis in traditional Chinese medicine, and indicated that tongue-coating microbiome could be a potential non-invasive biomarker, which might be suitable for long-term monitoring of gastritis.

**Electronic supplementary material:**

The online version of this article (10.1007/s13238-018-0596-6) contains supplementary material, which is available to authorized users.

## Introduction

Gastritis, which is a worldwide problem, is defined as an inflamed condition of the gastric mucosa (Price, 1991; Stolte and Meining, 2001; Owen, 2003; Rugge et al., 2007). Gastritis development is a multistep and multifactorial process (Guo et al., 2017). The stage of gastritis can be divided into superficial gastritis, atrophic gastritis, intestinal metaplasia and dysplasia (Correa, 1992; Correa and Piazuelo, 2012). Long-term studies have confirmed that the development of gastritis increases the risk of gastric cancer (Sipponen et al., 1985; Filipe et al., 1994; Miehlke et al., 1998; Meining et al., 2002; Ohata et al., 2004; Song et al., 2015). Gastritis diagnosis in clinical practice relies primarily on endoscopy and histological examination (Dixon et al., 1996; Rugge et al., 2007), which are invasive procedures that cannot be done frequently. Hence, monitoring and controlling at regular intervals with non-invasive methods are highly demanded in the prevention and treatment of gastritis. Therefore, it is important to find biomarkers associated with the occurrence and development of gastritis.

Bacteria were shown to contribute to gastritis when Barry Marshall and Robin Warren found in 1984 that *Helicobacter pylori* (HP) in the stomach played an important role in chronic gastritis (Marshall and Warren, 1984). However, it was reported that approximately 25% of chronic gastritis patients were not infected by *Helicobacter pylori*, suggesting that some other bacteria or factors, which are capable of causing inflammation, are involved (Jonkers et al., 1997). Since then, a series of studies based on different cohorts in stomach have confirmed the role of bacteria other than *Helicobacter pylori* in gastric lesions (Sjostedt et al., 1985; Sahay et al., 1995; Li et al., 2009; Hakalehto et al., 2011; Aviles-Jimenez et al., 2014; Eun et al., 2014; Schulz et al., 2016; Ferreira et al., 2017; Sohn et al., 2017). The Firmicutes phylum and the *Streptococcus* genus were found to exhibit increased abundance in gastritis patients compared to normal controls (Li et al., 2009). Analyses of gastric mucosa microbial changes of superficial gastritis (SG), atrophic gastritis (AG), intestinal metaplasia (IM) and gastric cancer (GC) found that gastric microbial composition and interaction shifted, indicating that gastritis development is associated with shifting human microbiota (Aviles-Jimenez et al., 2014; Eun et al., 2014; Coker et al., 2017).

Among diseases in the digestive tract, gastritis status may be reflected in the coating of tongue, which is the initial part of the digestive tract. On the one hand, food and microbes transferred into the stomach could remain in the residue on the tongue coating. On the other hand, the gastroesophageal reflux of gastritis patients could bring materials from the stomach to the tongue coating. The association between difference in tongue coating microbiota and the occurrence of gastritis has been explored in several studies. Our previous work focused on the tongue coating of gastritis patients and initially found 123 and 258 species-level OTUs enriched in the tongue coating of gastritis with Cold Syndrome and that with Hot Syndrome, respectively. Moreover, tongue-coating images of healthy controls and gastritis patients were distinguishable (Jiang et al., 2012). Sun and colleagues confirmed that microbial components of tongue coating in chronic gastritis patients were different from those in normal controls based on 16S ribosomal RNA denatured gradient gel electrophoresis (Sun et al., 2013). Ye et al. found that *Bacillus* was present only in the yellow tongue coating of chronic erosive gastritis patients by using Illumina Miseq sequencing of the V4–V5 region of the 16S ribosomal RNA gene (Ye et al., 2016). Furthermore, for traditional Chinese medicine (TCM), tongue appearance is a major indicator of physical status including that of the stomach. Our previous work also confirmed that tongue images could be used to classify gastritis patients and healthy volunteers and to classify gastritis patients with different TCM subtypes (Kanawong et al., 2012). However, as far as we know, there are no systematical analyses based on metagenomic sequencing to reveal the tongue coating microbiome variation associated with the occurrence and development of gastritis. This study focused on whether the gastric condition is reflected in the tongue-coating microbiome, which might be objective, non-invasive and suitable for long-term monitoring.

In this study, we collected the tongue coating samples of 78 gastritis patients and 50 healthy subjects. Metagenomic analysis revealed that the variation in tongue-coating microbiota was associated with the occurrence and development of gastritis. A network including 21 tongue-coating species that differentiated the tongue-coating microbiomes of gastritis patients and healthy controls was identified. Pathways such as microbial metabolism in diverse environments, biosynthesis of antibiotics and bacterial chemotaxis were up-regulated in gastritis patients. Furthermore, the abundance of *Campylobacter concisus* was detected to be associated with the precancerous cascade of gastritis. To test whether *Campylobacter concisus* existed in the stomach, we detected the *Campylobacter concisus* in tongue coating and gastric fluid in a validation cohort of 38 gastritis patients by quantitative polymerase chain reaction (qPCR). The study showed that tongue coating microbiota could potentially be a non-invasive biomarker, which might be objective and suitable for long-term monitoring.

## Results

### Patient and healthy control characteristics

**Figure 1:**
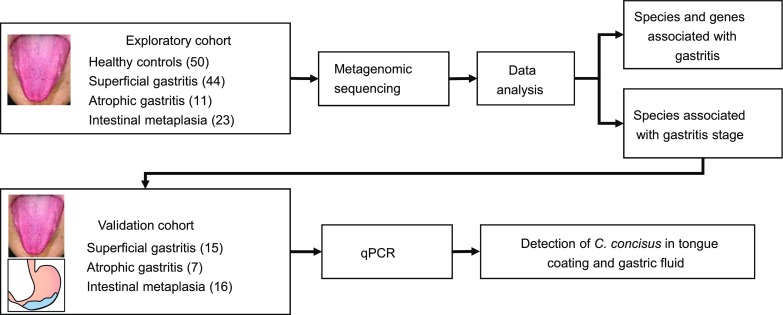
**Graphic summary of the study design**

In an exploratory cohort, 78 gastritis patients received an endoscopic examination were enrolled. Gastritis patients were divided into 3 groups according to gastric precancerous cascade, including superficial gastritis, atrophic gastritis and intestinal metaplasia, based on histopathology. Fifty healthy volunteers with no complaints of stomach discomfort were recruited as normal controls (Tables 1 and S1). Tongue images and tongue-coating samples were collected from all participants. For patients, the clinical symptoms were also recorded (Table S2). In a validation cohort, 15 superficial gastritis, 7 atrophic gastritis and 16 intestinal metaplasia patients were recruited. Metagenomic sequencing was conducted in the exploratory cohort to determine the tongue-coating microbiota associated with gastritis. *Campylobacter concisus* was tested in tongue coating and gastric fluid in the validation cohort by qPCR (Fig. 1).

**Table 1:** Demographic characteristics of healthy controls and gastritis patients.

Demographic variable	Characteristics	Normal controls	Gastritis patients			
			Total	Superficial gastritis	Atrophic gastritis	Intestinal metaplasia
Sample size		50	78	44	11	23
Age	Mean ± SD	44 ± 15.6	48 ± 13.5	46 ± 14.5	47 ± 12.3	55 ± 11.5
Sex	Male/Female	23/27	31/47	16/28	3/8	10/13
HP	Positive/Negative	NA	23/55	16/28	2/9	5/18

### Phylogenetic and gene profiles of tongue-coating microbiomes in controls and patients

**Figure 2:**
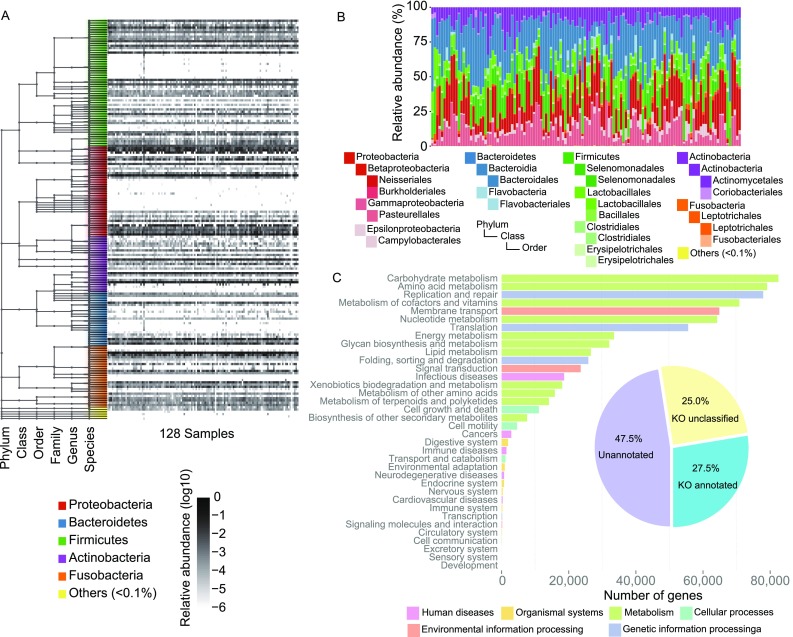
**Taxonomy and gene profiles of the tongue-coating samples**. (A) The relative abundance of species identified in tongue-coating samples, ordered by the taxonomy tree. (B) Relative abundance of major phyla across tongue-coating samples. (C) Gene counts annotated to different KO functions

Metagenomic sequencing was performed on tongue-coating samples, generating approximately 24–211 million reads per sample. We denoted this database as Meta-Tongue database. Using a pipeline similar to the Human Microbiome Project (HMP), 176 species were obtained from all tongue coating samples. These species were classified into 12 phyla, 21 classes, 35 orders, 56 families and 87 genera (Fig. 2A and Table S3). Variations of microbial composition at the phylum level between individuals could be seen (Fig. 2B). *De novo* sequence assembly and gene clustering on tongue coating samples identified ~1.9 M non-redundant putative genes. The genes were annotated to 5,377 KOs and classified according to functions (Fig. 2C and Table S4).

Comparing the taxonomy of normal controls and gastritis patients, we found that 149 species were shared in normal controls and gastritis patients, and 17 species were observed only in normal controls, whereas 10 species were observed in gastritis patients. Considering the stage of patients, 8 species, 1 species and 1 species were observed only in superficial gastritis, atrophic gastritis and IM patients, respectively. Comparing the microbial constitution of HP-positive and HP-negative patients, 143 species were shared in both groups, whereas 14 species and 6 species existed only in HP-positive and HP-negative patients, respectively (Fig. S1). Considering the abundance of microbes at the phylum level, Proteobacteria, Firmicutes, Fusobacteria, Actinobacteria and Bacteroidetes dominated the tongue coating microbiota. Among which, Fusobacteria was significantly higher in patients (*P* = 0.003, Wilcoxon rank-sum test followed by FDR correction), whereas others had not significant differences between two groups (Fig. S2).

### Tongue-coating species as a potential biomarker associated with gastritis

**Figure 3:**
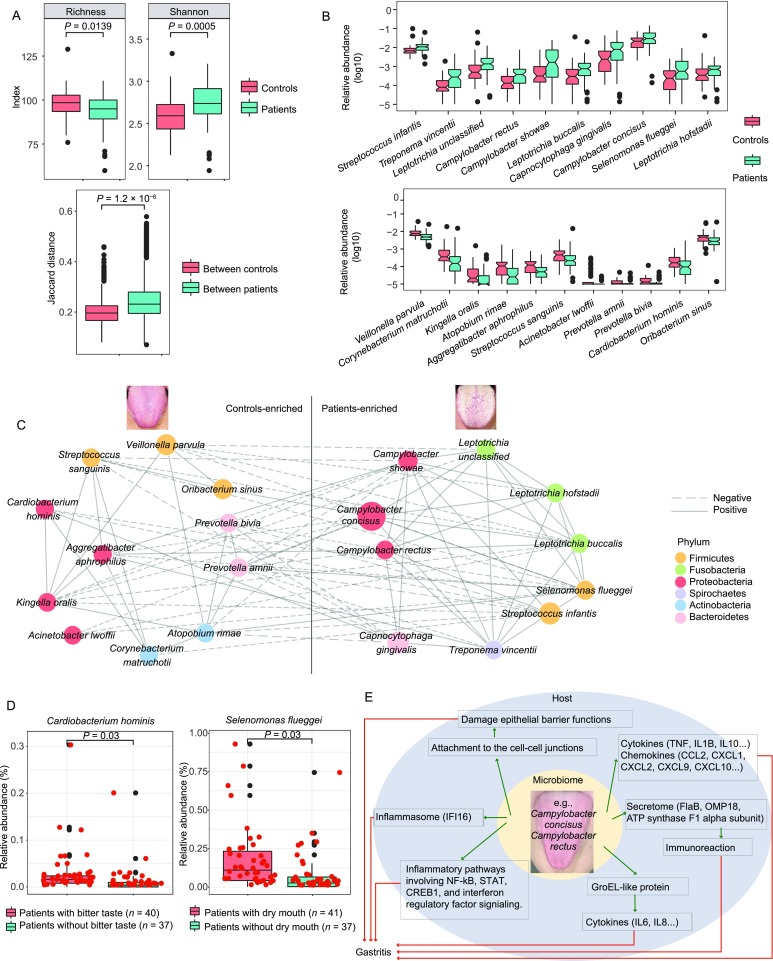
**Species with significantly different abundance in tongue coating of gastritis patients and normal controls**. (A) Top, richness and Shannon index in normal controls and gastritis patients. Down, Jaccard distances between normal controls and between gastritis patients. (B) Top, significantly decreased abundance of species in gastritis patients. Down, significantly increased abundance of species in gastritis patients. Boxes describe the interquartile range (IQR). Red and green boxes represent the abundance of the species in normal controls and gastritis patients, respectively. The y-axis represents the log of the abundance of the corresponding species in all samples. Species from the left to the right were arranged in ascending order of *P* value. (C) A correlation network of species associated with gastritis. All nodes represented species that are potential biomarkers associated with gastritis. They were classified into two groups including controls-enriched species and patients-enriched species. The size of the nodes indicates the mean abundance of the species among all samples. The color of the nodes represents the taxonomy assignment, with nodes of the same color belonging to the same phylum. Edges between nodes represent the correlation between the abundance of two species. Two nodes are linked if the Spearman correlation test shows a *P*-value < 0.05. Solid lines represent a positive correlation, while dashed lines represent a negative correlation. The width of the edges is proportional to the correlation strength, and wide line indicates strong correlation. (D) Species whose abundance is associated with particular symptoms. Statistical comparison by Wilcoxon rank-sum test followed by false discovery rate correction. (E) A schematic diagram showing the potential functions of some species enriched in patients that have a predicted gastritis association

In terms of alpha diversity, the species richnesses in gastritis patients were significantly lower than those of normal controls (*P* = 0.01, Wilcoxon rank-sum test), indicating a lower number of species in gastritis patients. The Shannon indexes were significantly higher than those of normal controls (*P* = 0.0005, Wilcoxon rank-sum test), indicating a more uniform species distribution in gastritis patients (Fig. 3A). The alpha diversities of HP-positive and HP-negative patients did not show a significant difference (Fig. S3). In terms of beta diversity, the Jaccard distances between gastritis patients were higher than those between normal controls, indicating that patients’ samples were highly dissimilar (Fig. 3A). There was no difference of Jaccard distances between HP-positive patients and those between HP-negative patients (Fig. S4). After Wilcoxon rank-sum test followed by FDR correction, no species were found significantly different between HP-positive and HP-negative patients. After comparing patients with different spicy preferences (23 prefer spicy food, 32 do not eat spicy food, 23 no obvious preference), no species were found to be significantly different among three groups (*P* > 0.5, Wilcoxon rank-sum test followed by FDR correction).

The Wilcoxon rank-sum test followed by FDR correction was used to characterize the significance of differences in tongue-coating microbiota abundance between the normal controls and gastritis patients. Finally, 21 species were found to be significantly different in abundance between the two groups (*P* < 0.05): 11 species including *Veillonella parvula*, *Corynebacterium matruchotii*, *Kingella oralis*, *Atopobium rimae*, *Aggregatibacter aphrophilus*, *Streptococcus sanguinis*, *Acinetobacter lwoffii*, *Prevotella amnii*, *Prevotella bivia*, *Cardiobacterium hominis* and *Oribacterium sinus* were decreased in gastritis patients, whereas 10 species including *Streptococcus infantis*, *Treponema vincentii*, *Leptotrichia unclassified*, *Campylobacter rectus*, *Campylobacter showae*, *Capnocytophaga gingivalis*, *Leptotrichia buccalis*, *Campylobacter concisus*, *Selenomonas flueggei* and *Leptotrichia hofstadii* were increased in gastritis group (Fig. 3B). These 21 species were defined as tongue-coating species associated with gastritis. To explore whether these tongue-coating species can distinguish gastritis patients from normal controls, hierarchical clustering of the abundance of these species in tongue coating samples from gastritis patients and normal controls was performed (Fig. S5). The clustering result showed that the gastritis patients group and normal controls group can be mainly separated, indicating that tongue-coating microbes may be potential biomarkers for gastritis.

A correlation network was constructed to assess the potential relationship between these 21 tongue-coating species (Fig. 3C). Controls-enriched species were significantly decreased in gastritis patients’ tongue coating, while patients-enriched species were significantly increased in gastritis patients’ tongue coating. Patients-enriched species had a stronger correlation with each other than controls-enriched species (*P* = 0.005, Wilcoxon rank-sum test), suggesting that patients-enriched species affected the host by interacting with each other and playing similar roles. Furthermore, the abundances of some species were also correlated with symptoms. *Cardiobacterium hominis* was higher in patients with bitter taste, and *Selenomonas flueggei* was higher in patients with dry mouth (Fig. 3D).

After exploring the potential functions of these tongue-coating species in gastritis by literature mining, we found some species enriched in patients that had the potential to induce inflammation and immune response in the host (Fig. 3E). *Campylobacter concisus* preferentially attached to cell-cell junctions, which led to damage of epithelial barrier functions (Man et al., 2010a, b). *Campylobacter concisus* could induce expression of cytokines and chemokines such as tumor necrosis factor (*TNF*), interleukin 1 beta (*IL1B*), interleukin 10 (*IL10*), C-C motif chemokine ligand 2 (*CCL2*), C-X-C motif chemokine ligand 1 (*CXCL1*), C-X-C motif chemokine ligand 2 (*CXCL2*), C-X-C motif chemokine ligand 9 (*CXCL9*), C-X-C motif chemokine ligand 10 (*CXCL10*), the assembly of inflammasome interferon gamma inducible protein 16 (IFI16) and activate the key inflammatory pathways involving nuclear factor kappa B (NF-kB), signal transducer and activator of transcription (STAT), cAMP responsive element binding protein 1 (CREB1) and interferon regulatory factor signaling (Man et al., 2010a, b; Kaakoush et al., 2015). Members of the *Campylobacter concisus* secretome including flagellin B (FlaB), ATP synthase F1 alpha subunit and outer membrane protein 18 (OMP18) were able to stimulate an immunoreaction (Kovach et al., 2011). Furthermore, the GroEL-like protein, which could induce the secretion of interleukin 6 (IL6) and interleukin 8 (IL8), could be secreted by *Campylobacter rectus* (Hinode et al., 1998). These findings showed the potential functions of species that had a predicted gastritis association.

### Differential abundance of tongue-coating microbial genes between controls and patients

To explore the variation in tongue-coating microbial functions in gastritis samples, genes of tongue-coating microbes were analyzed. After comparing the genes annotated to Kyoto Encyclopedia of Genes and Genomes (KEGG) between normal controls and gastritis patients by Wilcoxon rank-sum test followed by FDR correction, we found that 878 genes were significantly different between normal controls and gastritis patients (*P* < 0.05). Among these, 519 genes were significantly increased in gastritis patients (Table S5), including DedD protein, threonine aldolase and N-acetylmuramoyl-l-alanine amidase. Moreover, 359 genes including adenosylcobinamide hydrolasewere, NADP-dependent alcohol dehydrogenase, putative metalloprotease and glyoxylate reductase were significantly decreased in gastritis patients (Table S6).

**Figure 4:**
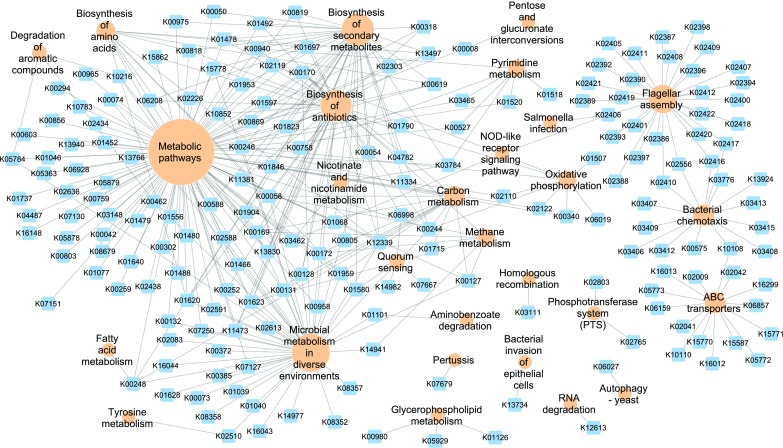
**Up-regulated tongue-coating microbial genes and enriched pathways**. Blue nodes represent KEGG KOs, and yellow nodes represent KEGG pathways. The edge between two nodes indicates that the gene was in the pathway. The size of the yellow node is proportional to the connection degree, i.e., pathways with a higher number of up-regulated genes exhibit larger nodes

In order to reveal the functions that up-regulated genes played, enrichment analysis was conducted by Fisher’s exact test followed by FDR correction. Finally, 519 up-regulated genes enriched in 28 pathways (*P* < 0.05) (Fig. 4). Pathways such as metabolic pathways, microbial metabolism in diverse environments, biosynthesis of secondary metabolites, biosynthesis of antibiotics, flagellar assembly, bacterial chemotaxis, ABC transporters, carbon metabolism and biosynthesis of amino acids were found to be enriched in up-regulated genes, indicating a variation of microbial functions in gastritis patients.

### *Campylobacter concisus* is associated with gastritis stages, and can be detected in gastric fluid

**Figure 5:**
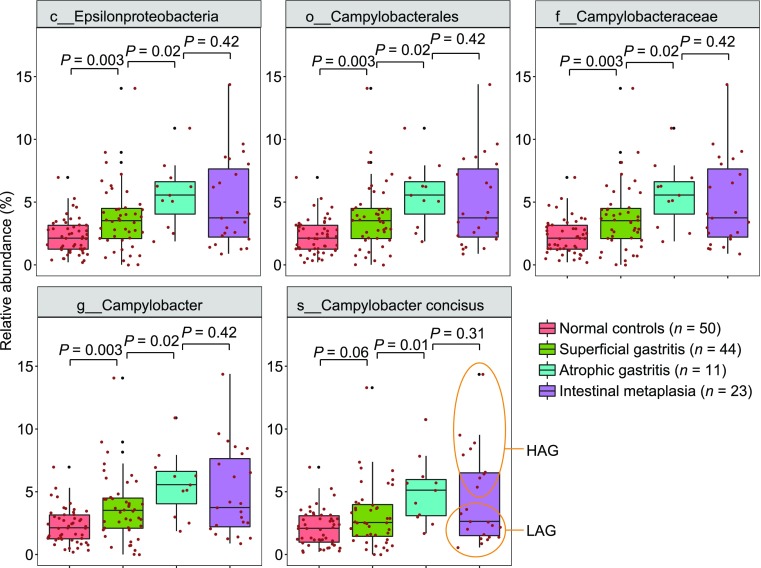
**The abundance of** ***Campylobacter concisus*** **during the precancerous cascade**. Boxes describe the interquartile range (IQR). The y-axis represents the relative abundance of the corresponding species in all samples. Statistical comparison by Wilcoxon rank-sum test

To assess the differences of patients in different stages of gastric precancerous cascade, the abundances of tongue-coating microbes were compared in four groups including normal controls, superficial gastritis, atrophic gastritis and intestinal metaplasia. This division indicated the development from normal status to the transformation from gastritis to precancerous lesions. Finally, the abundance of *Campylobacter concisus*, together with the abundances of the class, order, family, and genus it belongs to, were found to have clear association with gastritis stages (*P* = 0.02, Cochran-Armitage test). The abundance of *Campylobacter concisus* in superficial gastritis, atrophic gastritis and intestinal metaplasia was higher than that in normal controls. In addition, the abundance of *Campylobacter concisus* increased during precancerous cascade (Fig. 5). The Cochran-Armitage test for trend showed that there was a significant association between the abundance of *Campylobacter concisus* and the precancerous cascade (*P* = 0.02), which was more significant when using only the first three stages (normal controls, superficial gastritis and atrophic gastritis, *P* = 0.004).

In the intestinal metaplasia group, the samples clearly clustered into two subclasses according to the abundance of *Campylobacter concisus*. We termed the subclass with high abundance as the “high abundance group” (HAG) and another subclass as the “low abundance group” (LAG). The HAG samples showed a distinct increasing trend together with the health and other two gastritis stages groups (Cochran-Armitage test for trend, *P* = 3.8 × 10^−5^). This indicated that high heterogeneity exists in the intestinal metaplasia stage and there may be subgroups with specific characteristics such as TCM or western medicine phenotypes, which requires further studies in large samples.

After analysis of the potential relationships between *Campylobacter concisus* and clinical symptoms, we found that gastritis patients with dry mouth had a higher abundance of *Campylobacter concisus* in tongue coating than others (Fig. S6).

We also tested whether *Campylobacter concisus* existed in the stomach. Gastric fluid samples from 38 gastritis patients with matched tongue-coating samples were collected as a validation cohort. We performed qPCR to measure the abundance of *Campylobacter concisus* (Table S7). *Campylobacter concisus* can be detected in both tongue-coating samples and gastric fluid samples.

## DISCUSSION

Evidence from decades of recent long-term clinical studies and epidemiological material indicates that non-resolving inflammation has strong relationships with tumorigenesis (Carl Nathan, 2010). At least 15% of malignant tumors worldwide, especially tumors in the digestive system, respiratory system and urogenital system, are derived from non-resolving inflammation (Lisa M. Coussens, 2002). The occurrence of gastric cancer has a strong correlation with gastritis (Ohata et al., 2004). Therefore, it would be very meaningful to find biomarkers for the occurrence and development of gastritis.

Our previous study has found that gut microbiome and molecular networks are associated with the process from inflammation to cancer of colorectal cancer (Liang et al., 2014), with some inflammation-cancer related microbes validated by later studies (Kesselring et al., 2016; Zitvogel et al., 2018). In this paper, we proposed tongue-coating microbes as potential biomarkers associated with gastritis including the gastric precancerous cascade, which might be objective, non-invasive and suitable for long-term monitoring. Many researchers have revealed a correlation between oral cavity microbiota and disease in other regions of the body. James et al. compared the salivary microbiota between pancreatic cancer patients and normal controls and found that *Streptococcus infantis* and *Campylobacter concisus* were increased in pancreatic cancer patients (Farrell et al., 2012). The increase in *Streptococcus infantis* and *Campylobacter concisus* was also observed in the tongue coating of gastritis patients in our results, indicating that these potential biomarkers may also be a reflection of other disease states. A low diversity was found in tongue coating microbiota in gastritis patients, which was consistent with findings in other diseases. A low diversity in the gut was linked to obesity and inflammatory bowel disease (Turnbaugh et al., 2008; Qin et al., 2010).

Some species of the genus *Campylobacter* are shown to be involved in human disease, of which *Campylobacter jejuni* and *Campylobacter coli* are the most common (Man, 2011; O Brien, 2017). *Campylobacter jejuni* and *Campylobacter coli* are able to cause gastrointestinal illness including enteritis (Allos, 2001; Tam, 2003). *Campylobacter concisus* has been found to exist in most parts of the human digestive tract, including the oral cavity, esophagus, stomach and colon (Macfarlane et al., 2007; Zhang et al., 2010; Mukhopadhya et al., 2011), and has been repeatedly proven to be associated with gastrointestinal disease. Previous studies mostly focused on the higher isolation incidence of *Campylobacter concisus* in patients with inflammatory bowel disease (IBD) (Engberg et al., 2005; Newell, 2005; Man et al., 2010; Kaakoush et al., 2011; Mukhopadhya et al., 2011; Kaakoush and Mitchell, 2012; Kaakoush et al., 2014; Zhang, 2014; Deshpande et al., 2016). *Campylobacter concisus* is also found to be linked with Barrett’s esophagus (Macfarlane et al., 2007). Researchers focusing on microbiota in the stomach fluid showed high transcriptional active of *Campylobacter concisus* (von Rosenvinge et al., 2013), indicating that *Campylobacter concisus* may play an important role in the stomach. Our study characterized the association between the abundance of *Campylobacter concisus* in tongue coating and the occurrence and development of gastritis. The abundance of *Campylobacter concisus* in the stomach did not show significant correlation with diseases stages. There is heterogeneity in the gastritis samples, which makes it difficult to show significant correlation in small sample cohort. There might be subgroups with specific characteristics such as TCM or western medicine phenotypes, which require further studies in large samples.

There are cheaper and convenient ways for gastritis diagnosis such as endoscopy and histological examination in clinical practice, however, they are invasive. Biomarkers from tongue coating microbiome based on tongue diagnosis could provide beneficial complement for gastritis diagnosis, from the non-invasive, individualized and long-term monitoring aspects. Furthermore, in traditional Chinese medicine, tongue coating can reflect the health status of human body. Our work provided the biological evidence of tongue diagnosis. We found that species associated with gastritis had potential correlation with symptoms including dry mouth and bitter taste. In addition, *Campylobacter concisus* and *Campylobacter rectus* were recorded to upregulate genes such as immune factors, cytokines and CCL2 (Kaakoush et al., 2015), which were associated with the Hot Syndrome of gastritis in TCM (Li et al., 2007; Li et al., 2013). Especially, TNF which can be induced by *Campylobacter concisus* is associated with bitter taste (Feng et al., 2015), which is an important Damp Heat phenotype related to gastritis in traditional Chinese medicine. These findings showed a slight hint that tongue coating species such as *Campylobacter concisus* may have potential association with Hot Syndrome, which includes status such as “Shang-huo”, Damp Heat, Yin-deficiency, and so on in TCM.

In conclusion, we have demonstrated the variation of tongue-coating microbiota in gastritis patients by metagenomic sequencing, identified potential biomarkers for gastritis including precancerous cascade. Our work takes a step toward a potential non-invasive biomarker for gastritis, which might be objective and suitable for long-term monitoring. Furthermore, before using tongue-tongue microbiomes as biomarker for larger samples, more studies are required to reveal the influence of food intake and the geographical areas of people. It has been known that *Helicobacter pylori* is an important factor in gastritis and has been found associated with some gastritis sub-types as well as gastric cancer in many studies (McColl, 2010). Gastritis is actually an “umbrella term” for a number of diseases including those induced by *Helicobacter pylori* and by other factors (Sugano et al., 2015; Suzuki and Mori, 2015). Patients without *Helicobacter pylori* infection may also have gastritis including superficial, atrophic or even gastric cancer (Genta and Sonnenberg, 2015; Pogoriler et al., 2015; Horiuchi et al., 2016; Overby et al., 2017). In future research on larger samples, taking precise *Helicobacter pylori* information into consideration in the study of gastritis sub-types and stages may reveal more specific biomarkers for the diagnose of gastritis and gastric cancer.

## MATERIALS AND METHODS

### Sample collection

For the exploratory cohort, 99 patients who received an endoscopic examination in Beijing Dongzhimen TCM Hospital, Beijing Xiyuan TCM Hospital and China-Japan Friendship Hospital were recruited. Endoscopic examinations were performed, and the clinical symptoms were recorded. Biopsy specimens were selected from the antrum. The histological assessment was done by two experienced pathologists following clinical guidelines according to “the updated Sydney System” (Dixon et al., 1996). The inclusion criteria were a confirmed diagnosis of gastritis according to histological examination. Autoimmune gastritis patients were excluded. The *Helicobacter pylori* infection for all patients was identified by pathological examination. Healthy volunteers were recruited from Tsinghua University during their annual physical examination in the hospital. Seventy-six healthy people who reported no complaints of stomach discomfort in the past 5 years and also confirmed no gastritis in the examination were enrolled. The exclusion criteria for both patients and healthy volunteers consisted of the use of glucocorticoids and antibiotics for the past 3 months. Tongue images and tongue coating samples were collected from all participants. After quality control of metagenomic sequencing, 167 samples including 93 patients and 74 controls passed the acceptance criteria. Samples with contamination and patients without histopathological results were then filtered. Finally, 78 patients and 50 controls were retained for further analysis. Patients were divided into different stages of gastric precancerous cascade according to the histopathological results. This study mainly focused on people living in Beijing.

For the validation cohort, 38 patients who received an endoscopic examination in the China-Japan Friendship Hospital were recruited with the same inclusion and exclusion criteria for the exploration cohort. Samples were different from that in exploratory cohort. Tongue-coating and gastric fluid samples were collected. Histological examination results and clinical symptoms were recorded.

### Specimen collection, DNA extraction and preservation

Tongue-coating swabs were used to collect tongue-coating samples of participants before consumption of breakfast and water. The tongue was scraped from the root to the tip 30 times by simultaneously rolling the swab, the swab was placed into an RNase-free Eppendorf tube with 1 mL of phosphate-buffered saline (PBS), and the swab was agitated in order to wash out the tongue coating. The above step was repeated twice with a new swab and new Eppendorf tube to ensure that the tongue coating was sufficiently collected. After collection of tongue coating, the tubes were centrifuged at 5,000 ×*g* for 5 min. Supernatant and sediment were preserved separately in sterile tubes and stored at −80 °C until analysis. DNA of microbiota in the samples was extracted following the user guide of a modified version of the MO-BIO PowerSoil DNA Isolation Kit (MO-BIO Laboratories, Inc., Carlsbad, CA, USA).

### Library construction and sequencing

The sequencing library was constructed using a modified version of the NEBNext Ultra DNA library Prep Kit (New England Biolabs, Ipswich, MA, USA) protocol. We constructed barcoded, paired-end libraries with an insert size of ~500 bp for each sample. Four samples were designed to be sequenced in each lane. Samples were randomly assigned to different sequence lanes. Extracted DNA (200 ng) was used for library construction for each sample. Samples were sequenced in two batches. Samples in the first batch (172 samples) were sent to BGI Shenzhen for sequencing. After quality determination, libraries passing quality control were sequenced with the Illumina HiSeq 2000 platform. The read length was set to 90 bp. Samples in the second batch (3 samples) were sequenced in Tsinghua University with the Illumina HiSeq 2500 platform. The read length was set to 100 bp.

### Quality control, assembly and gene prediction

A modified version of fastx_barcode_splitter in the FASTX-Toolkit (http://hannonlab.cshl.edu/fastx_toolkit/) was used to decode the barcoded samples of each lane with the parameter “—mismatches 2”. We used the pipeline “assemble_revise_predict_genes_with_hg19_screen” in the “runMOCAT.pl” of the MOCAT V1.3 (http://vm-lux.embl.de/~kultima/MOCAT/) (Kultima et al., 2012) toolkit for quality control, assembly and gene (ORF) prediction. The configured file of MOCAT was similar to that used by Li et al. (2014).

Sequences after quality control were clean reads of high quality without human reads. To ensure better reliability, only samples with more than 15 M clean reads were used for subsequent analysis. A total of 167 samples passed the entire quality control (Table S8).

### Taxonomy profiling

We used MetaPhlAn Version 1.7.8 (http://huttenhower.sph.harvard.edu/metaphlan) (Segata et al., 2012) to calculate the taxonomy abundance table for each sample. MetaPhlAn aligns short reads directly to the customized prokaryote database, which was constructed by extracting clade-specific marker genes from the NCBI genome database with the phylogenetic information of the NCBI taxonomic tree. Abundances were calculated based on read counts of corresponding marker genes normalized by gene length and sequencing depth. We used clean reads as inputs, and the parameters were “–bt2_ps very-sensitive, -t ‘rel_ab’”.

### Construction and annotation of Integrated Tongue Gene Catalogue (ITGC)

We first constructed a “Meta-Tongue Gene Catalogue” (MTGC) for the Meta-Tongue dataset. We merged all the ORFs from 167 high quality samples and did a redundancy removal according to their sequence similarity using CD-HIT (Li and Godzik, 2006; Fu et al., 2012) v4.6.1 (http://weizhongli-lab.org/cd-hit/). The parameters we used in CD-HIT were “-G 0 -M 0 -T 4 -c 0.95 -aS 0.9 -n 8 -B 1”. To construct a more comprehensive catalogue, we collected HMP dorsum samples (Huttenhower et al., 2012) and used a same pipeline as above to build an “HMP Tongue Gene Catalogue” (HTGC). We then merged MTGC and HTGC using CD-HIT with same parameters, and got the final Integrated Tongue Gene Catalogue (ITGC).

We mapped ITGC genes to NCBI nr database (created in 2012.2.28 by NCBI) with blastp (Altschul et al., 1990) in the Blast 2.2.29+ toolkit. We used “-evalue 1e-5 -outfmt 6” in our study. The outputs from blastp were processed with MEGAN (Huson et al., 2007) v5.7.1 (http://ab.inf.uni-tuebingen.de/software/megan5/) with parameters “maxMatches=100 minScore=50.0 maxExpected=0.01 topPercent=10.0 minSupport=1 minComplexity=0.0 useMinimalCoverageHeuristic=false paired=false useIdentityFilter=false”.

### Gene and function profiling

For each sample, we mapped its clean paired-end reads to ITGC genes, calculated gene abundances and function abundances according to the mapping results. We used SOAPaligner v2.2.1 (http://soap.genomics.org.cn/soapaligner.html) with parameters “-r 2 -M 4 -l 30 -v 9” in our study. The ITGC gene relative abundances were calculated in a similar way with that in the work of Qin et al. (2012).

### Statistical analysis

To determine the differential abundance of species and genes between controls and patients, the Wilcoxon rank-sum test was used. *P*-values were adjusted with false discovery rate. The correlation between species was analyzed using Spearman’s correlation. The comparison of alpha diversity index and Jaccard distance was tested by Wilcoxon rank-sum test. The increasing trend of the abundance of *Campylobacter concisus* with gastritis stages was tested by Cochran-Armitage test. Samples in HAG were labeled as 1 and others were labeled as 0. Health and the three gastritis stages were labeled as 0, 1, 2, 3 separately.

### Detection of *Campylobacter concisus* by qPCR

Abundances of *Campylobacter concisus* in tongue coating and gastric fluid were assessed using quantitative PCR (qPCR). Universal 16S rDNA was used as internal reference for the estimation of all the microbes. The abundance of *Campylobacter concisus* was estimated using the proportion of *Campylobacter concisus* in all the microbes. qPCR was performed with TransStart Top Green Qpcr SuperMix.

## Electronic supplementary material

Below is the link to the electronic supplementary material.

Electronic supplementary material 1 (PDF 460 kb)

Electronic supplementary material 2 (XLSX 9138 kb)
