# Supplementary material for: Tongue coating microbiome as a potential biomarker for gastritis including precancerous cascade
Source: Protein Cell. 2018 Nov 26;10(7):496–509. doi: 10.1007/s13238-018-0596-6 (PMC6588651; doi:10.1007/s13238-018-0596-6)
Supplement: Supplementary file 1 — Electronic supplementary material 1 (PDF 460 kb) [file 13238_2018_596_MOESM1_ESM.pdf]

Supplementary Materials for

Tongue coating microbiome as a potential biomarker for gastritis including precancerous cascade

Jiaxing Cui*, Hongfei Cui*, Mingran Yang, Shiyu Du, Junfeng Li, Yingxue Li,

Liyang Liu, Xuegong Zhang[†], and Shao Li[†]

* These authors contributed equally to this work.

† Corresponding author: S.L., shaoli@tsinghua.edu.cn, and X.Z., zhangxg@tsinghua.edu.cn

This PDF file includes:

Fig. S1. Venn diagram of species composition in different groups.
Fig. S2. Tongue-coating microbial composition at the phylum level between normal controls and gastritis patients.
Fig. S3. Comparison of alpha diversity in HP-positive patients and HP-negative patients.
Fig. S4. Comparison of beta diversity in HP-positive patients and HP-negative patients.
Fig. S5. Clustering of tongue-coating samples from gastritis patients and normal controls.
Fig. S6. The abundance of Campylobacter concisus in patients with and without dry mouth.

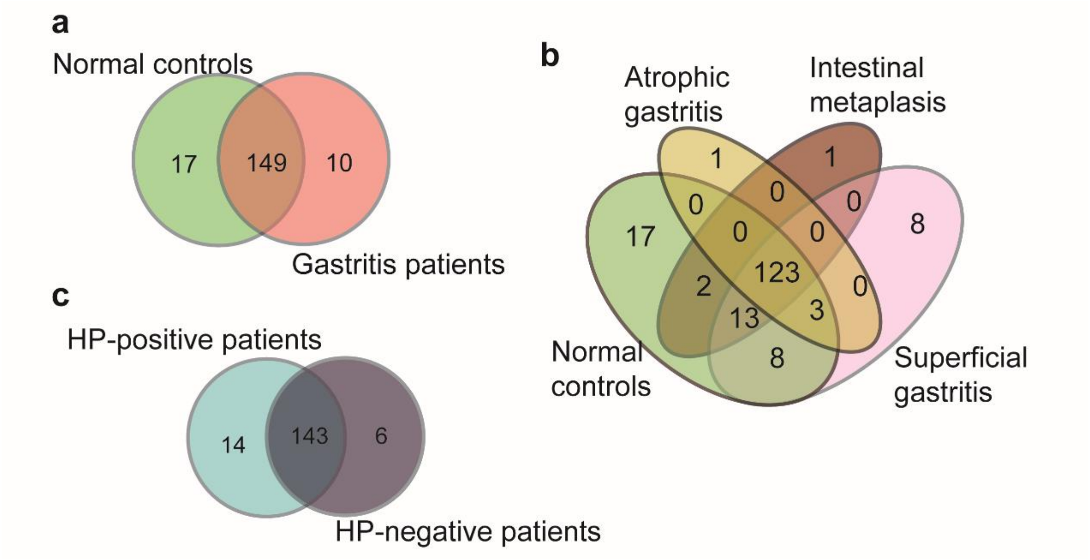

Supplementary Fig. S1. Venn diagram of species composition in different groups. (**a**) Normal controls and gastritis patients. (**b**) Normal controls, superficial gastritis patients, atrophic gastritis patients and IM patients. (**c**) HP-positive patients and HP-negative patients.

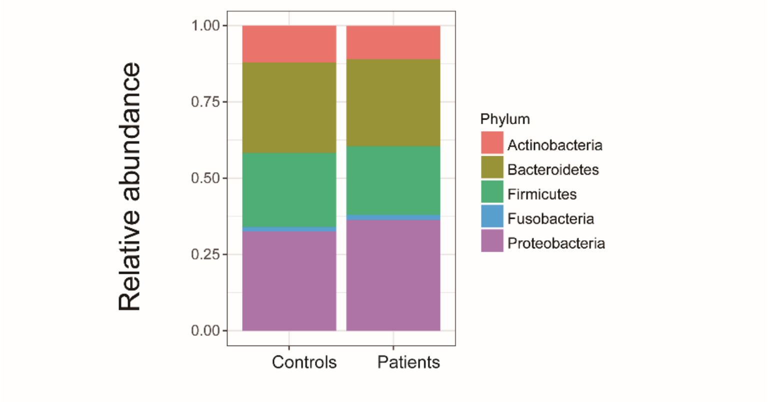

Supplementary Fig. S2. Tongue-coating microbial composition at the phylum level between normal controls and gastritis patients. Phyla whose relative abundance >0.1% were shown.

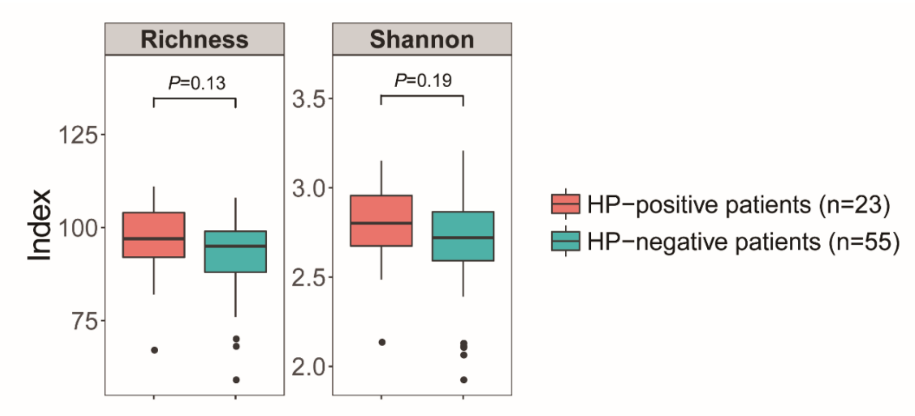

Supplementary Fig. S3. Comparison of alpha diversity in HP-positive patients and HP-negative patients. Statistical comparison by Wilcoxon rank-sum test.

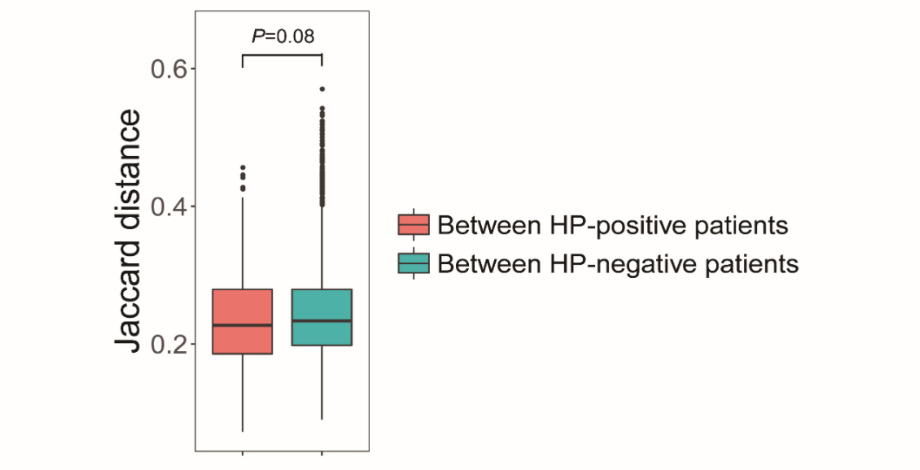

Supplementary Fig. S4. Comparison of beta diversity in HP-positive patients and HP-negative patients. Statistical comparison by Wilcoxon rank-sum test.

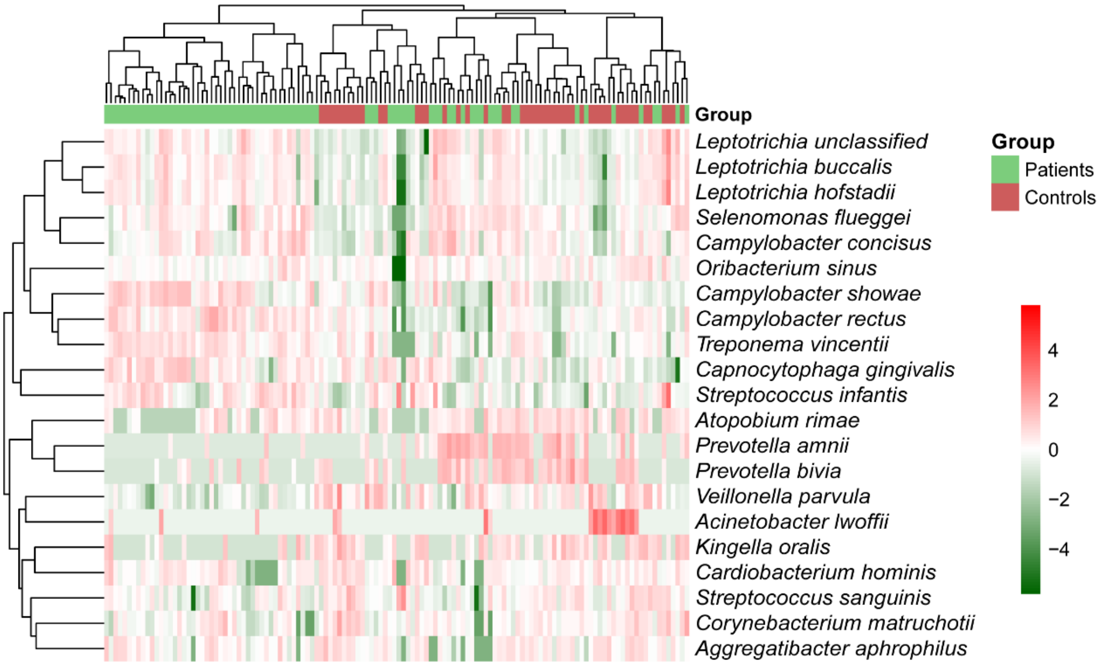

Supplementary Fig. S5. Clustering of tongue-coating samples from gastritis patients and normal controls. Rows represent species associated with gastritis. Columns represent all samples. The color in each cell indicates the relative abundance (log10) of the species in the corresponding sample. The values in row direction were centered and scaled. The distance measure used in clustering columns was the Pearson correlation.

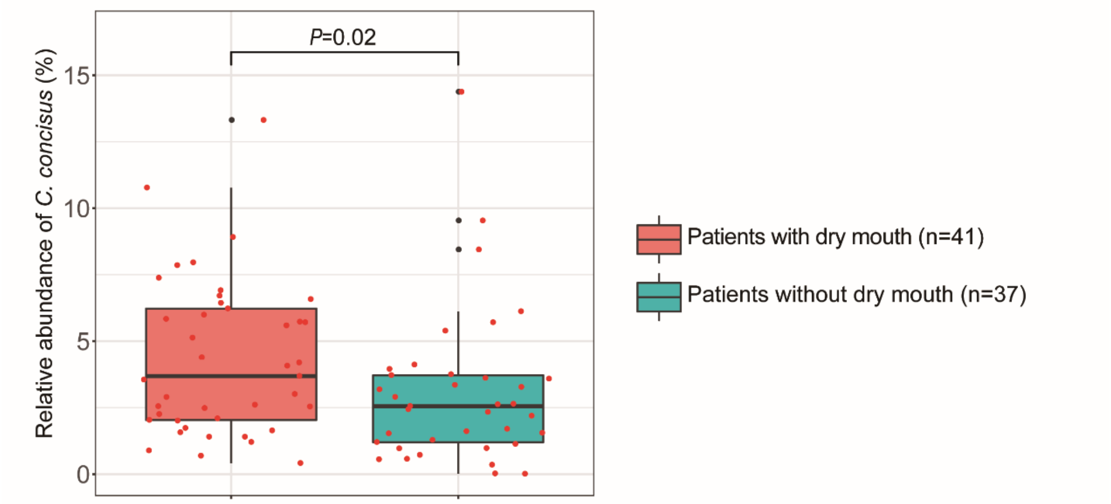

Supplementary Fig. S6. The abundance of *Campylobacter concisus* in patients with and without dry mouth. Statistical comparison by Wilcoxon rank-sum test.
